# Accurate Parameter Estimation for Unbalanced Three-Phase System

**DOI:** 10.1155/2014/539420

**Published:** 2014-08-04

**Authors:** Yuan Chen, Hing Cheung So

**Affiliations:** Department of Electronic Engineering, City University of Hong Kong, Kowloon, Hong Kong

## Abstract

Smart grid is an intelligent power generation and control console in modern electricity networks, where the unbalanced three-phase power system is the commonly used model. Here, parameter estimation for this system is addressed. After converting the three-phase waveforms into a pair of orthogonal signals via the *α* *β*-transformation, the nonlinear least squares (NLS) estimator is developed for accurately finding the frequency, phase, and voltage parameters. The estimator is realized by the Newton-Raphson scheme, whose global convergence is studied in this paper. Computer simulations show that the mean square error performance of NLS method can attain the Cramér-Rao lower bound. Moreover, our proposal provides more accurate frequency estimation when compared with the complex least mean square (CLMS) and augmented CLMS.

## 1. Introduction

Due to the increasing demand for electricity and the finite supply of nonrenewable energy sources, electrical power generation systems have faced a huge challenge. In order to improve the efficiency and reliability and to reduce the cost of electricity network, the concept of smart grid [1] is proposed, which can utilize the renewable and sustainable resources such as wind and solar energies. Traditional grid is a star network including a central point (e.g., power generation station) and leaf nodes (e.g., user terminals), whereas the smart grid is a mesh network whose nodes can act as both users and generators. In smart grid, because of this role conversion and the operations in terminals [2], frequency and amplitude variations exist and they can cause many serious problems such as loss of synchronism, power system stabilization, and equipment connection [3]. As a result, it is important to monitor the variations via accurately estimating the corresponding parameters [4].

It is common to use the unbalanced three-phase power system [5] for modeling in smart grid applications. Conventional estimators exhibit poor behavior applied for the three-phase system directly, because they work well only for the single-phase signal, which cannot truly characterize the unbalanced system [6]. Although we can perform estimation in each phase separately, accurate results may not be obtained because fixed phase displacement does not hold. Nevertheless, by making use of the *αβ*-transformation [7], the three-phase waveforms can be mapped into a pair of in-phase and quadrature signals. Based on this model, a number of approaches for frequency estimation have been proposed, including the complex least mean square (CLMS) [8] and augmented complex least mean square (ACLMS) [9] methods. Nevertheless, both estimators only focus on finding the frequency of the unbalanced system. In this work, we contribute to the development of an optimal estimator for the frequency, phase, and amplitudes from the orthogonal signals.

The rest of this paper is organized as follows. In Section 2, the problem is formulated and then nonlinear least squares (NLS) estimator is devised. We apply the Newton-Raphson scheme to solve the corresponding nonlinear optimization problem where algorithm initialization and global convergence are examined. Computer simulations are included in Section 3, which show that the mean square error (MSE) performance of NLS method can attain the optimum benchmark of the Cramér-Rao lower bound (CRLB) in the presence of white Gaussian disturbances and its superiority over the CLMS and ACLMS algorithms in frequency estimation is demonstrated. Finally, conclusions are drawn in Section 4.

## 2. Proposed Method

### 2.1. Development and Convergence Analysis

The discrete-time observations of the unbalanced three-phase power system are modeled as [5]:

(1) $$\begin{array}{c} v_{a}\left[n\right]=V_{a}\cos\left(\omega n+\phi \right)+\xi_{a}\left[n\right],\\ v_{b}\left[n\right]=V_{b}\cos\left(\omega n+\phi -\frac{2\pi }{3}\right)+\xi_{b}\left[n\right],\\ v_{c}\left[n\right]=V_{c}\cos\left(\omega n+\phi +\frac{2\pi }{3}\right)+\xi_{c}\left[n\right], \end{array}$$

where *V*_*a*_, *V*_*b*_, and *V*_*c*_ are the inequivalent amplitudes of different phase components, *ω* = *Ω*/*F*_*s*_ is the discrete frequency with *Ω* and *F*_*s*_ being the voltage frequency in radian and sampling frequency in Hz, respectively, and *ϕ* is the initial phase. The nominal value of *Ω* is Ω* = 100*π* (or 120*π*) rads^−1^. According to [10], the noise terms *ξ*_*a*_[*n*], *ξ*_*b*_[*n*], and *ξ*_*c*_[*n*], are independent and identically distributed additive white Gaussian noise sequences with same variance *σ* ^2^. The task is to find the unknown parameters, namely, *ω*, *ϕ*, *V*_*a*_, *V*_*b*_, and *V*_*c*_. In this study, we apply the *αβ*-transformation [7] on (1) to achieve accurate parameter estimation. The transformed signals, denoted by *v*_*α*_[*n*] and *v*_*β*_[*n*], are computed as

(2) $$\begin{array}{c} \left[\begin{array}{c} v_{\alpha }\left[n\right]\\ v_{\beta }\left[n\right] \end{array}\right]=\sqrt{\frac{2}{3}}\left[\begin{array}{ccc} 1 & -\frac{1}{2} & -\frac{1}{2}\\ 0 & \frac{\sqrt{3}}{2} & -\frac{\sqrt{3}}{2} \end{array}\right]\left[\begin{array}{c} v_{a}\left[n\right]\\ v_{b}\left[n\right]\\ v_{c}\left[n\right] \end{array}\right]. \end{array}$$

Based on (1)-(2), *v*_*α*_[*n*] and *v*_*β*_[*n*] can also be expressed as

(3) $$\begin{array}{c} v_{\alpha }\left[n\right]=s_{\alpha }\left[n\right]+q_{\alpha }\left[n\right],\\ v_{\beta }\left[n\right]=s_{\beta }\left[n\right]+q_{\beta }\left[n\right], \end{array}$$

where

(4) sα[n]=(Acos⁡(ϕ)−Bsin(ϕ))cos⁡(ωn) −(Asin(ϕ)+Bcos⁡(ϕ))sin(ωn),sβ[n]=(−Bcos⁡(ϕ)+Csin(ϕ))cos⁡⁡(ωn) +(Bsin(ϕ)+Ccos⁡(ϕ))sin(ωn),

with

(5) $$\begin{array}{c} A=\frac{\sqrt{6}}{12}\left(4V_{a}+V_{b}+V_{c}\right),\\ B=\frac{\sqrt{2}}{4}\left(V_{b}-V_{c}\right),\\ C=\frac{\sqrt{6}}{4}\left(V_{b}+V_{c}\right),\\ q_{\alpha }\left[n\right]=\sqrt{\frac{2}{3}}\left(\xi_{a}\left[n\right]-\frac{1}{2}\xi_{b}\left[n\right]-\frac{1}{2}\xi_{c}\left[n\right]\right),\\ q_{\beta }\left[n\right]=\frac{\sqrt{2}}{2}\left(\xi_{b}\left[n\right]-\xi_{c}\left[n\right]\right). \end{array}$$

Although both *q*_*α*_[*n*] and *q*_*β*_[*n*] contain *ξ*_*b*_[*n*] and *ξ*_*c*_[*n*], it is easy to show that the noise terms are uncorrelated; that is, *E*{*q*_*α*_[*n*]*q*_*β*_[*n*]} = 0 where *E* denotes the expectation operator, and they have identical variance *σ* ^2^.

Assuming that we have *N* samples for each channel, (3) can be written in matrix form as follows:

(6) $$\begin{array}{c} v=s+q, \end{array}$$

where

(7) $$\begin{array}{c} v={\left[\begin{array}{cccccccc} v_{\alpha }\left[1\right] & v_{\alpha }\left[2\right] & \cdots & v_{\alpha }\left[N\right] & v_{\beta }\left[1\right] & v_{\beta }\left[2\right] & \cdots & v_{\beta }[N] \end{array}\right]}^{T},\\ s={\left[\begin{array}{cccccccc} s_{\alpha }[1] & s_{\alpha }[2] & \cdots & s_{\alpha }[N] & s_{\beta }[1] & s_{\beta }[2] & \cdots & s_{\beta }[N] \end{array}\right]}^{T},\\ s=Gx,\\ G=\left[\begin{array}{cc} H & 0\\ 0 & H \end{array}\right],\\ H=\left[\begin{array}{cc} \cos\left(\omega \right) & \sin\left(\omega \right)\\ \cos\left(2\omega \right) & \sin\left(2\omega \right)\\ \vdots & \vdots \\ \cos\left(N\omega \right) & \sin\left(N\omega \right) \end{array}\right],\\ x=\left[\begin{array}{c} A\cos\left(\phi \right)-B\sin\left(\phi \right)\\ -A\sin\left(\phi \right)-B\cos\left(\phi \right)\\ -B\cos\left(\phi \right)+C\sin\left(\phi \right)\\ B\sin\left(\phi \right)+C\cos\left(\phi \right) \end{array}\right],\\ q={\left[\begin{array}{cccccccc} q_{\alpha }[1] & q_{\alpha }[2] & \cdots & q_{\alpha }[N] & q_{\beta }[1] & q_{\beta }[2] & \cdots & q_{\beta }[N] \end{array}\right]}^{T}. \end{array}$$

Here, *T* denotes the transpose operator and** 0** is the *N* × 2 zero matrix. We see that **x** corresponds to the linear unknowns, while *ω* is the nonlinear unknown in (6). Employing NLS [11, 12], the estimates of *ω* and **x**, denoted by $\hat{\omega }$ and $\hat{x}$, are

(8) $$\begin{array}{c} \left\{\hat{\omega },\hat{x}\right\}=\arg\underset{\omega ,x}{\min\;}J\left(\omega ,x\right),\;\;J\left(\omega ,x\right)={\left(v-Gx\right)}^{T}\left(v-Gx\right). \end{array}$$

Based on the Newton-Raphson procedure, the updating rule for $\hat{\omega }$ is

(9) $$\begin{array}{c} {\hat{\omega }}^{\left(k+1\right)}={\hat{\omega }}^{\left(k\right)}-\frac{\nabla_{\omega }J\left({\hat{\omega }}^{\left(k\right)},{\hat{x}}^{\left(k\right)}\right)}{\nabla_{\omega }^{2}J\left({\hat{\omega }}^{\left(k\right)},{\hat{x}}^{\left(k\right)}\right)}, \end{array}$$

where

(10) $$\begin{array}{c} \nabla_{\omega }J\left(\omega ,x\right)=-{\left(G_{1}x\right)}^{T}\left(v-Gx\right),\\ \nabla_{\omega }^{2}J\left(\omega ,x\right)=x^{T}\left(G_{1}^{T}G_{1}\right)x+{\left(G_{2}x\right)}^{T}\left(v-Gx\right), \end{array}$$

with

(11) $$\begin{array}{c} G_{1}=\left[\begin{array}{cc} H_{1} & 0\\ 0 & H_{1} \end{array}\right],\\ H_{1}=\left[\begin{array}{cc} -\sin\left(\omega \right) & \cos\left(\omega \right)\\ -2\sin\left(2\omega \right) & 2\cos\left(2\omega \right)\\ \vdots & \vdots \\ -N\sin\left(N\omega \right) & N\cos\left(N\omega \right) \end{array}\right],\\ G_{2}=\left[\begin{array}{cc} H_{2} & 0\\ 0 & H_{2} \end{array}\right],\\ H_{2}=\left[\begin{array}{cc} \cos\left(\omega \right) & \sin\left(\omega \right)\\ 4\cos\left(2\omega \right) & 4\sin\left(2\omega \right)\\ \vdots & \vdots \\ N^{2}\cos\left(N\omega \right) & N^{2}\sin\left(N\omega \right) \end{array}\right]. \end{array}$$

Here, ${\hat{\omega }}^{\left(k\right)}$ and ${\hat{x}}^{\left(k\right)}$ are the estimates of *ω* and **x** at the *k*th iteration. Once we have ${\hat{\omega }}^{\left(k\right)}$, ${\hat{x}}^{\left(k\right)}$ is easily obtained from (8) as

(12) $$\begin{array}{c} {\hat{x}}^{\left(k\right)}={{\left(G^{T}G\right)}^{-1}G^{T}v|}_{\omega ={\hat{\omega }}^{\left(k\right)}}, \end{array}$$

where −1 denotes the matrix inverse. To start the algorithm of (9) and (12), we need ${\hat{\omega }}^{\left(0\right)}$. Noting that *ω* should be around its nominal value ω* = Ω*/*F*_*s*_, that is, *ω* ∈ [ω* − *τ*, ω* + *τ*] where *τ* is the maximum deviation from ω*, ${\hat{\omega }}^{\left(0\right)}$ is computed using grid search as follows. We assign *K* uniformly-spaced grid points in the range [ω* − *τ*, ω* + *τ*] where one of them is ${\hat{\omega }}^{\left(0\right)}$. For each possible candidate ${\hat{\omega }}^{\left(0\right)}$, we determine ${\hat{x}}^{\left(0\right)}$ according to (12). The pair $\left\{{\hat{\omega }}^{\left(0\right)},{\hat{x}}^{\left(0\right)}\right\}$ which gives the minimum value of *J*(*ω*, **x**) will be chosen as the initial guess for (9). In our study, the iterative algorithm is terminated when $|\left({\hat{\omega }}^{\left(k+1\right)}-{\hat{\omega }}^{\left(k\right)}\right)/\left({\hat{\omega }}^{\left(k+1\right)}\right)|<\epsilon$, where *ϵ* > 0 is a small tolerance constant, is reached. After obtaining $\hat{\omega }$, the NLS estimates of *ϕ*, *V*_*a*_, *V*_*b*_, and *V*_*c*_ are straightforwardly computed from $\hat{x}$ as

(13) ϕ^=tan⁡−1(x^3−x^2x^1+x^4),V^a=(62x^1−66x^3)cos⁡⁡(ϕ^) −(62x^2+66x^3)sin(ϕ^),V^b=(x^4−2x^2)cos⁡(ϕ^)+(63x^3−2x^1)sin(ϕ^),V^c=(x^4+2x^2)cos⁡(ϕ^)+(63x^3+2x^1)sin(ϕ^),

where ${\hat{x}}_{m}$ (*m* = 1,2, 3,4) denotes the *m*th element of $\hat{x}$.

Finally, we examine the convergence of (9). According to [13], global convergence with quadratic rate is guaranteed when $M|\omega -{\hat{\omega }}^{\left(0\right)}|<1$ is satisfied, where *M* is as follows:

(14) $$\begin{array}{c} M=\underset{\omega \in \{-\pi ,\pi \}}{\sup}\left|\frac{\nabla_{\omega }^{3}J\left(\omega ,x\right)}{\nabla_{\omega }^{2}J\left(\omega ,x\right)}\right|. \end{array}$$

To determine the value of *M*, we first relate **G**, **G**_1_, and **G**_2_ as follows:

(15) $$\begin{array}{c} G_{1}^{T}G_{1}+G_{2}^{T}G=cE,\\ G_{1}=N_{1}GF,\\ G_{2}=N_{2}G, \end{array}$$

where

(16) $$\begin{array}{c} E=\left[\begin{array}{ccc} 1 & 0 & 0\\ 0 & 1 & 0\\ 0 & 2 & 0 \end{array}\right],\\ F=\left[\begin{array}{cccc} 0 & -1 & 0 & 0\\ 1 & 0 & 0 & 0\\ 0 & 0 & 0 & -1\\ 0 & 0 & 1 & 0 \end{array}\right],\\ c=\frac{N\left(N+1\right)\left(2N+1\right)}{6},\\ N_{1}=\mathrm{diag}\left(\left[\begin{array}{cccccccc} 1 & 2 & \cdots & N & 1 & 2 & \cdots & N \end{array}\right]\right),\\ N_{2}=\mathrm{diag}\left(\left[\begin{array}{cccccccc} 1 & 2^{2} & \cdots & N^{2} & 1 & 2^{2} & \cdots & N^{2} \end{array}\right]\right). \end{array}$$

Based on (10)-(11), ∇_*ω*_^2^ *J*(*ω*, **x**) can be expressed as

(17) ∇ω2J(ω,x)=xT(cE−G2TG)x+xTG2T(v−Gx) ≥xTGTN2v−2xTGTN2Gx ≥xTGTN2v−2N2xT(GTG)x.

Equality holds if and only if *x*_1_^2^ + *x*_2_^2^ + 2*x*_2_ *x*_3_ = 0, *ω* = *mπ* with *m* = −1, −1/2, 0, 1/2, 1 and

(18) ∇ω3J(ω,x)=xTG1TN2v=xTFTGTN3v,

where **N**_3_ = diag⁡([1 2^3^ ⋯ *N* ^3^ 1 2^3^ ⋯ *N* ^3^]). Substituting (12) into (17)-(18) yields

(19) ∇ω2J(ω,x)≥vT(G(GTG)−1GT)N2v  −2N2vT(G(GTG)−1GT)v≥vTW1v,∇ω3J(ω,x)=vTW2v,

where

(20) $$\begin{array}{c} W_{1}=G{\left(G^{T}G\right)}^{-1}G^{T}\left(N_{2}-2N^{2}\right),\\ W_{2}=G{\left(G^{T}G\right)}^{-1}F^{T}G^{T}N_{3}. \end{array}$$

We can then write *M* as

(21) $$\begin{array}{c} M=\underset{\omega \in \{-\pi ,\pi \}}{\sup}\left\{\left|\frac{\nabla_{\omega }^{3}J\left(\omega ,x\right)}{v^{T}v}\right|\left|\frac{1}{\left(\nabla_{\omega }^{2}J\left(\omega ,x\right)\right)/\left(v^{T}v\right)}\right|\right\}=\left|\frac{\lambda_{2}}{\lambda_{1}}\right|, \end{array}$$

where *λ*_1_ and *λ*_2_ are the minimum eigenvalue of **W**_1_ and the maximum eigenvalue of **W**_2_, respectively. It is easily shown that *λ*_1_ = *N* ^3^ and *λ*_2_ = *N* ^2^. Hence *M* = *N*. As a result, if the initial estimate is chosen such that $N|\omega -{\hat{\omega }}^{\left(0\right)}|<1$ is satisfied, global solution will be obtained.

### 2.2. Cramér-Rao Lower Bound

Let the unknown parameter vector be ***θ*** = [*ω* *ϕ* *V*_*a*_ *V*_*b*_ *V*_*c*_]^*T*^. Then, the CRLB of $\hat{\theta }$ is obtained from the diagonal elements of the inverse of the Fisher information matrix, denoted by **F**(***θ***) [14]. The (*i*, *j*) entry of **F**(***θ***) is written as

(22) $$\begin{array}{c} {\left[F\left(\theta \right)\right]}_{i,j}={\left(\frac{\partial s}{\partial \theta_{i}}\right)}^{T}C^{-1}\left(\frac{\partial s}{\partial \theta_{j}}\right),\;i,j=1,\ldots ,5, \end{array}$$

where

(23) ∂s∂θi=[∂sα[1]∂θi∂sα[2]∂θi⋯∂sα[N]∂θi∂sβ[1]∂θi∂sβ[2]∂θi⋯∂sβ[N]∂θi]T,

with

(24) ∂sα[n]∂ω=−n(Acos⁡(ϕ)−Bsin(ϕ))sin(ωn) −n(Asin(ϕ)+Bcos⁡(ϕ))cos⁡(ωn),∂sβ[n]∂ω=n(Bcos⁡(ϕ)−Csin(ϕ))sin⁡(ωn) +n(Bsin(ϕ)+Ccos⁡(ϕ))cos⁡(ωn),∂sα[n]∂ϕ=(−Asin(ϕ)−Bcos⁡(ϕ))cos⁡⁡(ωn) −(Acos⁡(ϕ)−Bsin(ϕ))sin(ωn),∂sβ[n]∂ϕ=(Bsin(ϕ)+Ccos⁡(ϕ))cos⁡⁡(ωn) +(Bcos⁡(ϕ)−Csin(ϕ))sin(ωn),∂sα[n]∂Va=63(cos⁡(ϕ)cos⁡(ωn)−sin(ϕ)sin(ωn)),∂sβ[n]∂Va=0,∂sα[n]∂Vb=(612cos⁡⁡(ϕ)−24sin⁡(ϕ))cos⁡⁡(ωn) −(612sin(ϕ)+24cos⁡(ϕ))sin(ωn),∂sβ[n]∂Vb=(−24cos⁡⁡(ϕ)+64sin⁡(ϕ))cos⁡⁡(ωn) +(24sin(ϕ)+64cos⁡(ϕ))sin(ωn),∂sα[n]∂Vc=(612cos⁡⁡(ϕ)+24sin⁡(ϕ))cos⁡⁡(ωn) −(612sin(ϕ)−24cos⁡(ϕ))sin(ωn),∂sβ[n]∂Vc=(24cos⁡⁡(ϕ)+64sin⁡(ϕ))cos⁡⁡(ωn) +(−24sin(ϕ)+64cos⁡(ϕ))sin(ωn)

and **C** is the covariance matrix of the noise term which is **C** = *σ* ^2^ **I** with **I** denoting the 2*N* × 2*N* identity matrix. The (*i*, *j*) element can be simplified as

(25) $$\begin{array}{c} {\left[F\left(\theta \right)\right]}_{i,j}=\frac{1}{\sigma^{2}}\overset{N}{\underset{n=1}{\sum }}\left(\frac{\partial s_{\alpha }\left[n\right]}{\partial \theta_{i}}\frac{\partial s_{\alpha }\left[n\right]}{\partial \theta_{j}}+\frac{\partial s_{\beta }\left[n\right]}{\partial \theta_{i}}\frac{\partial s_{\beta }\left[n\right]}{\partial \theta_{j}}\right). \end{array}$$

## 3. Simulation Results

To assess the proposed estimator for the unbalanced three-phase power system, computer simulations have been conducted. The MSEs, $E\{{\left(\hat{\omega }-\omega \right)}^{2}\}$, $E\{{\left(\hat{\phi }-\phi \right)}^{2}\}$, $E\{{\left({\hat{V}}_{a}-V_{a}\right)}^{2}\}$, $E\{{\left({\hat{V}}_{b}-V_{b}\right)}^{2}\}$, and $E\{{\left({\hat{V}}_{c}-V_{c}\right)}^{2}\}$, and the mean frequency estimate, are employed as the performance measures. Comparisons with the CLMS and ACLMS algorithms as well as the CRLB are also made. We choose *Ω* = 101*π* rad/s and *F*_*s*_ = 5000 Hz, and hence *ω* = 0.0202*π* with ω* = 0.02*π*. The remaining parameters for (1) are assigned as *ϕ* = *π*/4, *V*_*a*_ = 1, *V*_*b*_ = 0.7, and *V*_*c*_ = 0.6. The maximum frequency deviation is *τ* = *π*/2500 which corresponds to ±2% difference from the nominal value, while the number of grid points is chosen as *K* = 30. When the condition *M* < 1/*τ* is satisfied, global convergence is ensured. The tolerance parameter for the frequency estimate update is *ϵ* = 10^−8^. All results are based on 1000 independent Monte Carlo runs.

**Figure 1:**
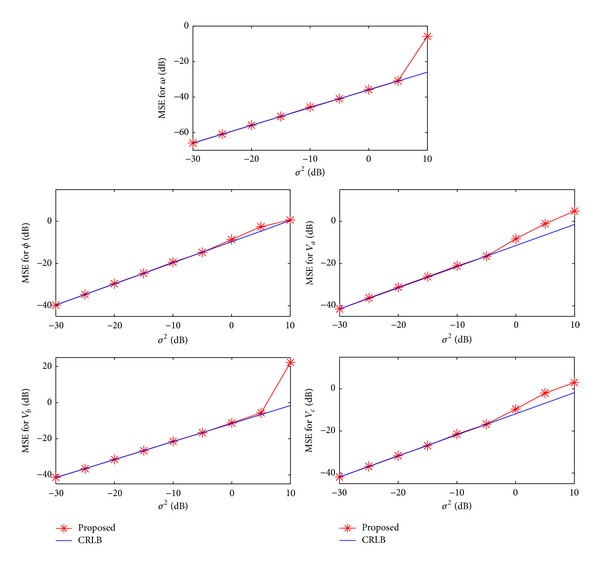
Mean square errors versus *σ* ^2^.

**Figure 2:**
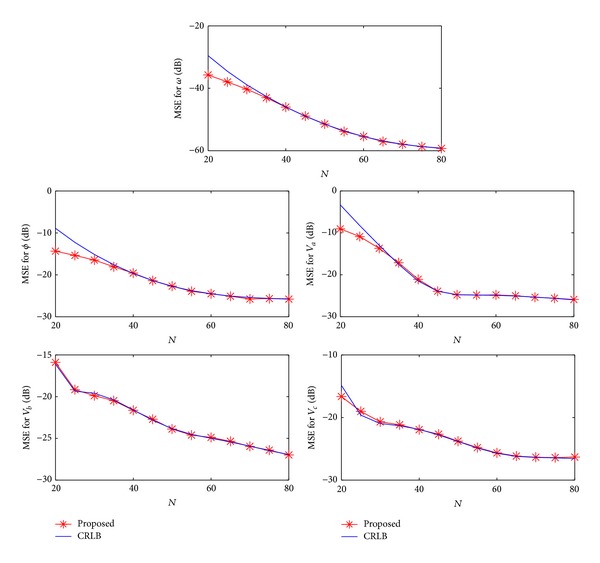
Mean square errors versus *N*.

First, we study the MSEs for *ω*, *ϕ*, *V*_*a*_, *V*_*b*_, and *V*_*c*_ versus *σ* ^2^ when the data length is assigned as *N* = 40, and the results are plotted in Figure 1. It is seen that, when *σ* ^2^ is sufficiently small, the MSE performance aligns with the CRLB, indicating the optimality of NLS estimator.  Figure 2 shows the MSEs versus *N* when the noise power is fixed at *σ* ^2^ = −10 dB. The high performance of the proposed scheme is again illustrated. Note that when *N* is small enough, the MSEs can be lower than the CRLB. It is because we have prior knowledge regarding the range of *ω*, but this information is not utilized in the CRLB derivation.

**Figure 3:**
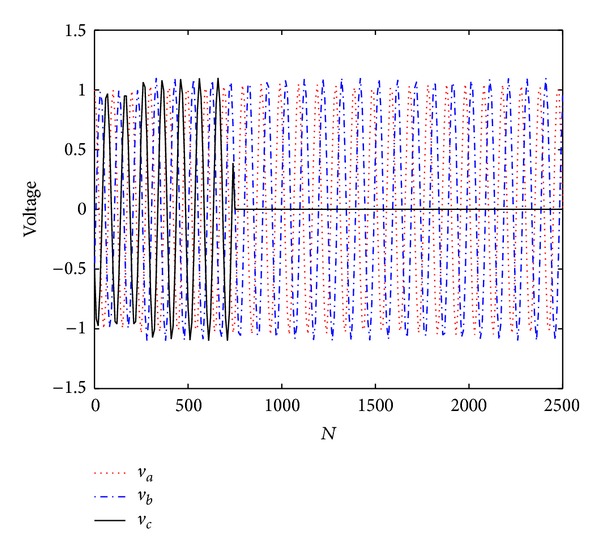
Three channel signals in unbalanced system.

**Figure 4:**
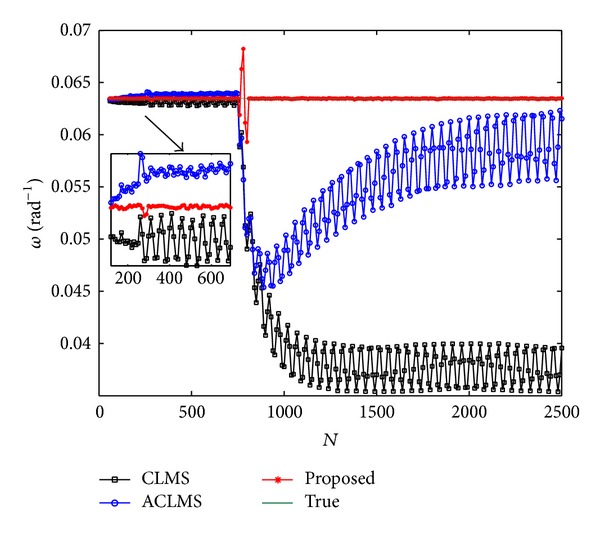
Mean estimated frequency versus *N* in unbalanced system.

Next, we compare the performance of the proposed estimator with the CLMS and ACLMS methods. The proposed method is a batch-mode method, so we make it adaptive by utilizing a sliding window with a length of *L* = 60 on the observations. That means if we receive *N* samples, the frequency is estimated by the former (*N* − *L* + 1) data. In this test, the noise power *σ* ^2^ is set to −20 dB. The frequency and phase parameters are the same with the former test, while the voltages are shown in Figure 3. When *N* < 250, *V*_*a*_ = *V*_*b*_ = *V*_*c*_ = 1. We add 0.05 to *V*_*a*_ with 0.1 to *V*_*b*_ and *V*_*c*_ from *N* = 250. Subsequently, *V*_*c*_ = 0 after *N* = 750.  Figure 4 shows the mean frequency estimates under the time-varying case and it is seen that the proposed method is superior to the CLMS and ACLMS algorithms in both the performance of estimating frequency and the robustness to abruptly change of voltage.

**Figure 5:**
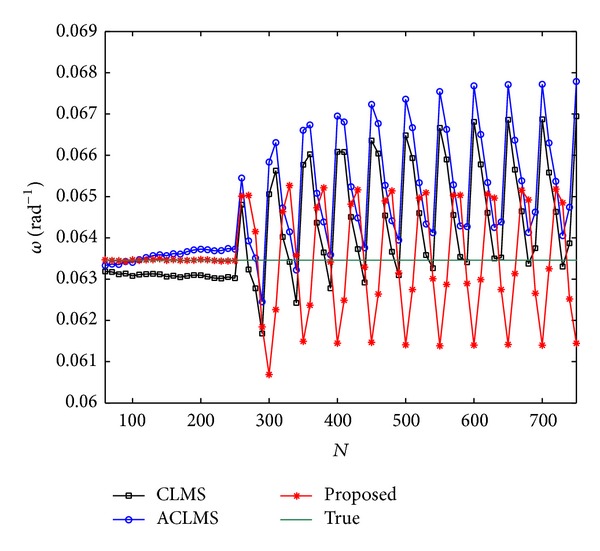
Mean estimated frequency versus *N* under harmonic contamination.

**Figure 6:**
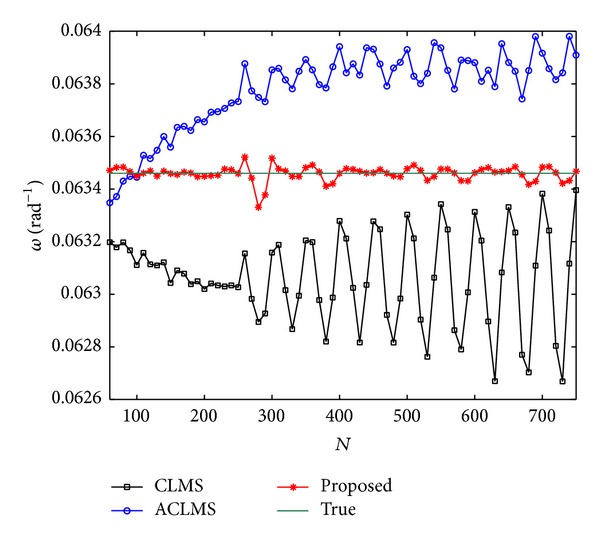
Mean estimated frequency versus *N* under amplitude variation.

Figure 5 shows the results when the observed data are contaminated by harmonics. We add a balanced 10% third harmonic and a balanced 5% fifth harmonic of the fundamental frequency *ω* to the system at *N* = 250. It can be seen that although three methods give fluctuating performance, our estimate always oscillates around the true frequency value of *ω* = 0.0202*π*. Finally, Figure 6 addresses the impact of amplitude oscillation. In this test, the voltages are set as *V*_*a*_ = 1 + 0.05sin(2*πn*/*F*_*s*_), *V*_*b*_ = 1 + 0.1sin(2*πn*/*F*_*s*_) and *V*_*c*_ = 1 + 0.15sin(2*πn*/*F*_*s*_) at *N* = 250. It is observed that our proposed method provides an estimate around the true value of *ω* = 0.0202*π* even when the amplitudes variation exist.

## 4. Conclusion

An accurate estimator for the unbalanced three-phase power system in the presence of additive Gaussian noise has been developed. The *αβ*-transformation is exploited to produce a pair of in-phase and quadrature signals from the three-phase waveforms, and then NLS cost function is constructed, where the frequency is the only nonlinear parameter. The Newton-Raphson scheme is employed to find NLS solution and its initialization and global convergence are studied. It is demonstrated that the MSE performance of the frequency, phase, and voltage estimates can achieve the CRLB and its mean frequency estimation accuracy is higher than that of the CLMS and ACLMS algorithms. A future work is to evaluate the developed algorithm using real three-phase power system measurements.
